# A Contemporary Evaluation on Posterior Direct Restoration Teaching among Undergraduates in Dental Schools in Malaysia

**DOI:** 10.3390/dj9100123

**Published:** 2021-10-19

**Authors:** Muhammad Syafiq Alauddin, Norazlina Mohammad, Azlan Jaafar, Faizah Abdul Fatah, Aimi Amalina Ahmad

**Affiliations:** 1 Department of Conservative Dentistry and Prosthodontics, Universiti Sains Islam Malaysia, Kuala Lumpur 55100, Malaysia; norazlina79@usim.edu.my (N.M.); drfaizah@usim.edu.my (F.A.F.); aimiamalina@usim.edu.my (A.A.A.); 2 Department of Periodontology and Community Oral Health, Universiti Sains Islam Malaysia, Kuala Lumpur 55100, Malaysia; drazlan_jaafar@usim.edu.my

**Keywords:** dental education, composite restoration, conservative dentistry, operative dentistry, undergraduate dental student, dentin bonding

## Abstract

There is a current trend to restore posterior teeth with composite resin due to increasing demands on natural tooth colour restoration and increased concern about the safety of amalgam restorations. The objective was to evaluate the current teaching of posterior direct restoration among restorative dental lecturers in Malaysia compared to available international literature. An online questionnaire, which sought information on the teaching of posterior restoration was developed and distributed to 13 dental schools in Malaysia. The response rate for the questionnaire was 53.8%. The most popular posterior restoration teaching methods among the respondents were lecture (95.7%), demonstration (87.0%) and problem-based learning (PBL) (73.9%), while continuous assessment and a practical competency test (82.6%) were the most popular assessment methods. Placing a hard setting calcium hydroxide and GIC base for deep cavity restored by composite restoration was taught in 79.2% of cases. The standard protocols for posterior composite restoration were incremental filling in deep cavity (87.5%), using circumferential metal bands with wooden wedge (91.7%), with a total etch system (95.8%), using a light emitting diode (LED) light curing unit (91.7%), finishing using water cooling (80%) and finishing with a disc (87.5%). Graduates from dental schools in Malaysia received similar theoretical, preclinical and clinical teaching on posterior restoration techniques, although there were variations in the delivery methods, techniques and assessments, pointing to a need for uniformity and consensus.

## 1. Introduction

Composite resin restoration is one of the dental practitioner choices besides traditional amalgam restoration. Rapid advances in the adhesive technology of the resin composites have resulted in them becoming the main choice to restore carious and traumatic posterior teeth [1,2]. After all, dental amalgams are a well-proven material with durability, excellence in maintaining structural integrity of the tooth-restoration complex in the long-term, as well as less technique sensitive steps being required for it. Nevertheless, due to its metallic greyish appearance and non-aesthetical optical properties, there is a current trend for metal free restorations. Moreover, there is a continuous issue with regards to its inability to bond to the tooth structure, marginal leakage and the high occurrence of secondary caries [3,4]. Evidence shows that a well restored composite resin restoration is able to provide a high survival and success rate of the posterior restoration [3,5,6]. It is also more aesthetically pleasing compared to the traditional amalgam restoration. With growing research and improvements in modern adhesive technology and science, contemporary resin composites are able to bond properly to the remaining tooth structure and reduce common risks associated with the resin composite restorations, such as polymerization shrinkage, marginal discolouration, bulk fracture, chipping of the restoration and microleakage [7,8,9,10], There is also a current trend worldwide in reducing use of mercury in dental practices. Amalgam restorations are known to emit mercury vapour during daily activities such as drinking hot water and eating, with potential, unfavourable toxicity side effects [11]. Furthermore, the current concept of a minimal invasive approach, which focuses more on collaborative work for conservative techniques, and treatment being introduced and advocated worldwide, means resin composites is seen as a more appropriate method. This is because dental amalgam restoration technically requires the removal of unnecessary, yet sound, tooth structure to provide more mechanical resistance and retention features. Thus, it is considered less conservative than resin composite restoration [12,13].

A plethora of components on competency-based pedagogy have been introduced during undergraduate clinical years, including training on posterior teeth restorations. Arguably the competency of undergraduate students in dental schools will predict the future outcome of these graduates in clinical practice after graduation, even though the competency of dental practitioners is commonly associated with experience and years of practice [14]. Therefore, it is of utmost importance that the faculty members and teaching staff constantly evaluate contemporary theoretical and clinical teaching methods, particularly in a routine dental procedure such as restoration of the posterior teeth. Undergraduate dental students should possess and exhibit acceptable competence in this field, particularly in biomaterial science and the use of modern contemporary materials, and use appropriate techniques to perform this procedure under the guidance of faculty members.

Several nationwide and regional surveys have been conducted over the past decades to evaluate the teaching of posterior restorations, especially for composite resin, and these have shown widely varying and notable differences in teaching programmes within and among the countries where the research had been conducted [15,16,17,18,19,20,21,22,23]. Apart from in Japan and Malaysia, these surveys provided limited data on the teaching of posterior composite resin restorations in Asian regions [24,25]. Taking into consideration the growing interest in metal free restorations and the need to homogenize and form coherency in the teaching of posterior restoration, the rationale of this study was to assess the current standard and approach for the teaching of posterior direct restorations on posterior teeth. The aim was to evaluate the trends, extent, nature and practice of contemporary teaching of posterior restoration to undergraduate dental students in Malaysia.

## 2. Materials and Methods

Ethical approval for this study was granted by the local institutional ethical committee [USIM/FPg-MEC/2016/No. (45)] for a cross-sectional and quantitative methodological approach conducted from January 2019 to May 2020.

A survey questionnaire was adopted, developed and then underwent minor modifications based on previous studies [20,25,26] (see Supplementary Figure S1). The questionnaire was then administered and distributed as an online survey via Google Form with the corresponding link being sent primarily via e-mail and then via WhatsApp to the heads and lecturers of the Operative/Conservative/Restorative Dentistry Department in all 13 dental schools in Malaysia that conduct dental degree programmes. The questionnaire was made up of 36 questions with a combination of open and closed questions. The majority were multiple choice questions with predefined answers that enabled the respondents to select more than one answer. The targeted respondents, who were Heads of Department Operative/Conservative/Restorative Dentistry and senior lecturers with 5 years or more experience in teaching posterior restoration to undergraduates, were initially given 12 weeks to complete the questionnaire. A second and third e-mail reminder was sent to participants who had not responded. They were informed that their participation in this research would remain anonymous and that the results would be confidential in that no individual and dental school would be identified in any preliminary findings, reports or publications.

The information sought from the respondents comprised of (a) the teaching technique and methodology of posterior restoration, including types of assessments, (b) the practice, nature and extent of the preclinical teaching, (c) the practice, nature and extent of the clinical teaching including relevant assessment techniques and (d) the contemporary restorative protocol taught and practiced for posterior teeth restoration with composite resins. Information derived from the questionnaires were entered into a Microsoft (R) Excel spreadsheet and descriptive data analysis was then performed to express results in terms of mean, range and total percentage in order to provide coherency of the reporting style associated with previous studies.

## 3. Result

### 3.1. Response Rate

From a total of 13 dental schools invited to collaborate in this study, seven schools responded to the questionnaire, a response rate of 53.8%. The findings stated are the responses given by the participating teachers from the dental schools, with responses to all or the majority of the questions.

### 3.2. Contemporary Teaching Methodology and Strategies

The theoretical pedagogical components including the mode of delivery, teaching aids and materials used to supplement the delivery of the core components and contemporary assessment methods involved in the teaching of posterior restoration in Malaysia are detailed in Table 1. According to the responses received, the most favourable teaching approaches for posterior tooth restoration were formal lectures (95.7%), followed by preclinical and clinical demonstrations (87%), problem-based learning (PBL) (73.9%), tutorials (69.6%) and seminars (47.8%). As for the teaching materials used to enhance learning activities, the majority of the respondents (65.2%) favoured the use of demonstrations through teeth models as compared to the distribution of validated instruction manuals which included clinical pro forma (43.5%). When it came to assessment methods to evaluate a student’s knowledge on posterior restoration throughout the undergraduate pro-gramme, a large number of the respondents (82.6%) preferred to conduct continuous assessments and clinical competency tests rather than written examinations (69.6%).

#### 3.2.1. Preclinical Simulation Laboratory/Phantom Head Teaching Programme

According to the results of this study, all the respondents agreed that the preclinical simulation laboratory practice should be a compulsory prerequisite for the students prior to treating patients during the clinical years (23 = (95.8%)). When asked about the types of posterior materials taught in the preclinical year, composite resin (*n* = 22 (95.7%)) was the most popular, then amalgam (*n* = 20 (87%)), followed by glass ionomer cement (GIC) (*n* = 8 (34.8%)) and resin modified GIC (*n* = 3 (13%)). The undergraduate students at most of the dental schools were provided with comprehensive manuals and instructions (*n* = 20 (81%)) on what was taught in class to aid them in the practice of preclinical simulation in the laboratory for posterior restorations, which mostly used both extracted natural teeth and artificial teeth (*n* = 12 (52.2%)). The schools unanimously agreed that students were required to complete a proximal amalgam and composite restoration as a prerequisite to start their clinical session. The average amount of preclinical time devoted to teaching composite resin placement during preclinical years was more than 24 h (*n* = 7 (30.4%)), followed by between 4 to 8 h (*n* = 7 (30.4%)), 12 to 16 h (*n* = 6 (26.1%)) and 20 to 24 h (*n* = 3 (13%)). During this time, the mean number of teeth required for the undergraduate to undergo training suggested by the respondents for preclinical simulation is shown in Table 2.

#### 3.2.2. Clinical Teaching Programme

The majority of the respondents stated that the teaching of amalgam restorations as a choice for posterior tooth restorative material was still relevant (*n* = 20 (83.4%)) except for 16.7% (*n* = 4) of respondents who responded otherwise. In this regard, the undergraduate students were required to complete amalgam restorations (*n* = 21 (87.5%)) and composite restorations (*n* = 22 (97.1%)), respectively, as part of their prerequisite requirement prior to the final examination during the 5th year of their undergraduate study.

Several assessment methods were used to evaluate the clinical competency of the undergraduate students. Among them were a clinical competency examination (*n* = 21 (87.5%)), followed by an objective structural clinical examination (OSCE) (*n* = 14 (58.3%)) and self-assessment (*n* = 10 (41.7%)). The least favoured assessments utilized by the dental schools were peer review/assessment (*n* = 5 (20.8%) (and viva voce examination (*n* = 7 (29.2%)).

### 3.3. The Management of Operatively and Partially Exposed Dentine

In the management of a moderate cavity depth restored using the amalgam restoration technique, no liner/base (45.8%) and hard setting calcium hydroxide (45.8%) and GIC base were preferred by respondents. The options selected were similar for composite resin restoration with no liner/base (50%) and hard setting calcium hydroxide (54.2%) considered as the most favourable options. In a deep cavity situation, the combination of hard setting calcium hydroxide liner and GIC base were the preferred options for both the amalgam (91.7%) and composite restoration (71.2%). As for a shallow cavity depth, none of the respondents reported teaching the use of cavity liner and/or base. The rest of the materials taught the use of liners, and the results are summarized in Table 3.

### 3.4. Restorative Materials Recommendation and Placed Sites on the Posterior Teeth

Maxillary and mandibular molars were the two most recommended and commonly placed sites for amalgam restorations, while maxillary and mandibular premolars were the most recommended and commonly placed sites for composite restorations. On the other hand, there were one (4.2%) and two (8.3%) respondents who recommended using GIC as an option to restore posterior sites. Further details and distribution with regards to the recommended and commonly placed restorative materials on posterior sites are summarized in Table 4.

### 3.5. Contemporary Operative Techniques Utilised in Dental Schools in Malaysia

#### 3.5.1. Moisture Control

All the respondents from various schools were in agreement that rubber dams were mandatory for composite restorations of posterior teeth (*n* = 24 (100%)). They were most commonly used to isolate the operative site for the placement of a posterior amalgam (*n* = 16 (66.7%)) and GIC (*n* = 18 (75%)), respectively. Besides the use of rubber dams, cotton rolls were considered as suitable alternative isolation tools for amalgam (*n* = 12 (50%)) and GIC (*n* = 8 (33.3%)) restorations.

#### 3.5.2. Beveling Technique

The most common technique taught to dental students was beveling the proximal box margins (*n* = 18 (75%)), followed by beveling the occlusal margin (*n* = 15 (62.5%)). Nevertheless, about 12.5% (*n* = 3) of the respondents reported not teaching any beveling techniques for posterior composite restorations.

#### 3.5.3. Adhesive

When asked about the adhesive bonding technique taught in dental schools in Malaysia, the majority of the respondents reported teaching the total etch system (*n* = 23 (95.8%)), whereas 45.8% (*n* = 11) of the respondents taught the self-etch technique for posterior composite resin restorations.

#### 3.5.4. Interproximal Matrix and Wedging Techniques

As for the isolation and wedge technique, a circumferential metal band with a wooden wedge (*n* = 22 (91.7%)) was the most common taught technique, followed by the sectional matrix system (*n* = 12 (50%)), sectional metal band with wooden wedge (*n* = 11 (45.8%)) and transparent matrix band with a light-transmitting wedge (*n* = 9 (37.5%)).

#### 3.5.5. Restorative Technique

According to the survey, the most common composite restoration technique taught for both deep and moderate cavity for composite restorations was incremental fill, with scores of 87.5% (*n* = 21) and 83.3% (*n* = 20), respectively. Nevertheless, 16.7% (*n* = 4) and 20.8% (*n* = 5) of the respondents reported teaching the bulk fill technique in deep and moderate cavity respectively.

#### 3.5.6. Light Curing Technologies

The majority of the respondents taught the students using a light-emitting diode (LED) curing light (*n* = 22 (91.7%)), and a small number of the respondents (*n* = 2 (8.3%)) reported still teaching the use of a “traditional” quartz-tungsten-halogen curing light for posterior composite restorations.

#### 3.5.7. Finishing Techniques

There was a considerable variety of finishing instruments used after completion of posterior restorations. Finishing discs and finishing strips were the most common finishing techniques taught, with scores of 87.5% and 75%, respectively. The utilization of diamond burs as one of the materials for finishing was the least taught in the dental schools (45.8%). Table 5 shows the variety of finishing technique/instruments taught for posterior restoration training in the dental schools in Malaysia.

## 4. Discussion

The overall participating response rate of dental schools, 53.8% (*n* = 7), in this study was among the lowest compared to previous surveys of a similar nature. The authors were not able to increase the response rate despite multiple attempts made to reach out to the selected dental schools. The initial part of the study focused on assessing the teaching strategies and methodology used for posterior restorations in Malaysia, as they had not been highlighted by other studies previously. The results of this study indicated that the respondents were almost in complete agreement that formal lectures and preclinical and clinical demonstrations formed a fundamental and integral part of teaching about posterior restorations. This has been reinforced in multiple studies and through surveys derived from dental students that revealed the teaching strategies used allowed comprehensive two-way interaction between the learners and the lecturers, with positive results achieved compared to other methods [27,28]. The majority (*n* = 19 (82.6%)) of the lecturers supplied a comprehensive instruction manual to their students. The materials were conventionally adopted from various guidelines that originated from manufacturers’ guidelines, recommendations from international professional bodies and societies, and fundamental core textbooks and literature in the respective fields. In assessing students, the two most popular assessment modalities reported were continuous assessments throughout the undergraduate years of study and practical competency tests. In assessing posterior restorations, the faculty members must first determine the desired learning outcome before selecting the assessment method, as the selection should be done according to the conventional outcome-based curriculum rather than the teacher-input-orientated traditional curriculum. The assessments conducted in the dental schools in Malaysia were made up of quizzes, mini examinations, clinical assessments and competency performance assessments designed in accordance with the four levels of Miller’s Pyramid of clinical competence and the affective, psychomotor and affective domains in Bloom’s Taxonomy of theoretical educational framework [29,30,31,32]. In this modern technological age, digital education implementation in posterior restoration teaching, such as e-learning and internet web-based education, is more desirable due to their practicality in facilitating the overall learning experience between students, lecturers, and faculty members. However, the findings for this are not reported in this study [33].

Prior to assessing dental students, the training carried out for them in various undergraduate dentistry programmes plays a major role at every step of the way. These students acquire the necessary theoretical knowledge at different stages of their studies and are assessed from time to time before they are finally ready for a clinical training placement mimicking the future roles as professional healthcare providers in a work setting. The fundamental challenge during this educational journey is to bridge these two training stages through gradual preclinical training. This integral training equips the undergraduates with the necessary skills to apply and integrate during theoretical and clinical practice prior to their clinical placement [34,35]. In this survey, the respondents unanimously agreed (100%) that the undergraduate dental students must complete preclinical training, as well as the restoration of proximal amalgam and composite, prior to any clinical training placements. Amalgam and composite resin restoration trainings had almost the same distribution in terms of the number of teeth (preclinical exercise) required to be completed during preclinical training, despite the prediction, based on previous studies, that amalgam restoration training might undergo marked reduction in the near future [16]. A clear majority of the restorative lecturers concluded that amalgam is still relevant to be taught in the dental curricular (83.4%). Apart from findings in Japan, the findings in this study were consistent with previous surveys which concluded that preclinical and clinical training of amalgam restoration is still relevant and considered as common practice in the UK, Germany, Austria and Switzerland [15,16,24,25,26,36]. Nevertheless, the Malaysia National Oral Health Survey (NOHSA) is on board to follow the call set by the global trend on phasing out amalgam, and it is perhaps beneficial to note that possible reduction on the preclinical training of amalgam restoration by using other suitable alternative materials will be implemented in the near future in the dental schools in Malaysia. However, other technical aspects, such as didactic training on repairing and maintaining existing restored amalgam of dental patients in Malaysia, should not be neglected [37,38]. It is regrettable that the authors were not able to compare the differences on preclinical training time dedicated between amalgam and composite resin restoration due to the inconsistent answers provided and the limited replies given for this section, as opposed to reports obtained from other studies [19,39].

It is noteworthy that one of the findings of this study indicated that the conventional teaching of placing a liner underneath an amalgam and composite restoration was considered not unusual in the dental schools in Malaysia. This is in contrast to the findings of other surveys of the same nature [15,18]. The routine practice of placing a liner underneath a composite restoration in a deep cavity is somehow controversial, and is considered an unnecessary step in moderately deep cavities [40]. The addition of a liner occupies part of the space for a composite resin bonding area, thus reducing the efficacy and effectiveness of the dentin bonding capability of the adhesive system. On another note, almost all the participating dental schools unanimously agreed to practicing the total etch system (95.8%), which, theoretically, is able to increase the sealing of dentine after effective selective etching [41,42]. The total etch system is able to ensure the effective removal of the smear layer after cavity preparation, which then promotes the ability of the adhesive resin system to permeate and seal the dentine. This prevents the need for lining materials to be used as additional protection [43]. Another finding in the study showed that the setting of calcium hydroxide, Ca(OH)2, was routinely taught as the only liner material, or to be used in combination with GIC. The biomechanical properties of Ca(OH)2 include its brittle nature and high solubility [41,42]. This has a negative impact upon polymerisation shrinkage of the composite resin, as it leaves the residual Ca(OH)2 exposed to undesired physical changes. When this happens, microleakage might follow, which then allows localised bacterial migration that might lead to recurrent caries. Anecdotal evidence that indicates a lining placement is able to inhibit hybrid layer degradation, and function as an antibacterial layer, has been refuted by previous evidence [44,45]. Rather, the traditional thought is that linings are placed in deep cavities for amalgam restorations to provide thermal insulation to the vital dentine and also for planned deep carious dentine remineralisation [41,43].

In this survey, the results indicated that the participants were more inclined towards teaching that the composite resin should be routinely placed on the premolar site while amalgam restorations should be mostly performed at the molar site. This is in line with previous studies that showed that amalgam has a high survival rate, with the majority of studies reporting more than 85% survival rate in an extensive cavities [46,47,48]. Moreover, composite resin restoration also has a low failure rate, with the majority of studies reporting an annual failure rate of less than 5%, thus making it a desirable material of choice for posterior teeth [1]. To ensure predictable and successful longevity of a posterior resin restoration, multiple studies concluded that there was a necessity to introduce caries preventive measures, or at least good caries control methods [3,5]. Nonetheless, despite the abundance of evidence showing the longevity of an amalgam restoration, necessary steps and plans are required by the operative dentistry community worldwide to support the global call to reduce the usage of amalgam as a restorative material.

Another finding from this study is in relation to the matrix and wedge technique used, with almost all respondents (91.7%) agreeing that the circumferential metal band with wooden wedge was commonly taught and practised in undergraduate dentistry programmes. This finding is consistent with surveys from other studies, which proves that the utilisation of this technique provided a more reliable posterior restoration outcome especially with Class II composite resin restorations [15,16,18,49,50,51]. Transparent matrices and stiff wedges, such as light-transmitting wedges, are nonrigid and are pliable in nature. This may cause possible mechanical deformation during clinical application especially during a composite resin restoration. Unlike amalgam, composite resin is unable to exert adequate physical force to hold the matrix system. This further complicates the conformity of the restoration in achieving proximal contact tightness with the adjacent teeth, which then results in a proximal overhang, open proximal contact and inappropriate contour [49]. Another point to note from this survey is that all the respondents from various backgrounds and schools agreed that rubber dams were considered mandatory for composite resin restorations. This finding was heavily reflected in this survey, and comparable to others of similar nature [15,16,18,20]. According to this survey, apart from cotton roll isolation, rubber dams were extensively used for amalgam and GIC posterior restorations as a precautionary measure. Composite resin restoration is a hydrophobic material and involves a technique-sensitive procedure. If it exposed to moisture intraorally, this might complicate successful bonding to the tooth structure. As such, common clinical conditions and situations, such as the presence of a deep subgingival margin, or the patient’s inability to tolerate rubber dams might be the most common contraindication for the application of rubber dams [18].

Some studies noted that confusion over additional beveling of the occlusal and proximal box margin in posterior restorations was influenced by the construction of beveling in anterior composite resin restorations [52]. Additional beveling in those locations can result in a number of disadvantages including improper marginal adaptation of the restoration at the tooth-restoration-matrix system area, the removal of unnecessary enamel structure that is paramount for effective bonding, thin composite resin residue at the beveling location of the cavosurface margin resulting in the possibility of a composite resin fracture or chipped restorations under physiological masticatory load in the future, and difficulties for the operators to identify between tooth tissues and composite resin restoration in future operative repair work. Regrettably, only 12.5% of the respondents thought that beveling in the proximal and occlusal margin was unnecessary, unlike those from the Spanish dental schools survey [16,18,23,24].

The majority of the restorative dentistry lecturers taught students to use a conventional light emitting diode (LED) curing light (97.1%). This finding represents one of the highest scores for this survey criterion as compared to the Japanese and Spanish dental school surveys [18,24]. Contemporary LED light curing units are, allegedly, able to provide more depth of cure, generate less heat, and have exceptional power and light intensity with less light exposure time, and are comparable, if not more effective, than traditional quartz tungsten halogen light curing units [53,54]. This survey also found that 37.5% of the respondents taught the bulk-fill composite resin technique in undergraduate dentistry programmes when compared to previous surveys of a similar nature [15]. Even though numerous studies showed that bulk fill composite resins are more controversial in their physical and biomechanical properties compared to other types and techniques of composite resin restorations, their feasibility and their major advantage of being able to reduce the chairside time makes them very attractive in dental practices [55,56,57,58].

In a recent publication by Sidhu, P. et al., in 2021, the study was similar in nature to the current study [59]. The respondents in the study included all the Heads of Conservative/Operative Dentistry Department, while in our study the questionnaire was sent to heads and lecturers who had a minimum of 5 years of teaching posterior restoration experience to undergraduates within the Operative/Conservative/Restorative Dentistry Department in all 13 dental schools in Malaysia. In the management of operatively and partially exposed dentine, both studies concluded that there should be no liners used in shallow cavities. According to this study, the combination of Ca(OH)2 and GIC was commonly taught and utilised as a lining material for amalgam (*n* = 22 (91.7%)) and resin composite (*n* = 19 (79.2%)) in deep cavities, while the study conducted by Sidhu, P. et al. 2021 received a score of 85% (*n* = 11). In the same study, the majority of the respondents selected rubber dams as mandatory tools for moisture control prior to resin composite placement (*n* = 11 (85%)) with the alternative tool being a cotton roll (*n* = 12 (92%)). This is similar to our findings [59]. Almost all respondents in both studies taught and utilized a conventional light emitting diode (LED) curing light and finishing techniques such as finishing discs and strips in the restoration of resin composites involving occlusal-proximal cavities. There was a slight notable discrepancy between the studies though. In our study, it was reported that the bulk fill technique was taught by 20.8% (*n* = 5) of the respondents, while there were no schools which practiced bulk fill teaching in the study carried out by Sidhu, P. et al. 2021 [59]. The specific teaching and learning activities in which the bulk fill technique was taught are not reported in our study.

Sidhu, P. et al. 2021 also reported on the contraindication of composite restoration placement at the posterior cavity, the contemporary composite materials and bonding systems used in dental schools, the fees charged by the dental schools for the restoration of a posterior cavity done by the students, and the teaching of indirect posterior composite resin restoration [59]. These were not included in our study The additional information obtained through the above-mentioned study is very much needed as it encourages a shift in the use of composite resins as a step towards a more modern way of dentistry, such as minimal intervention dentistry [59]. Nonetheless, this study was conducted with the aim of identifying the theoretical, didactic and clinical teaching used in the teaching and learning process, and covered the theoretical pedagogical component, teaching materials and common assessment types performed by dental schools in Malaysia. Findings on preclinical simulation programmes, and common types of assessment to evaluate clinical competency of the dental students, were also included. With both studies being conducted in Malaysia, it shows that there is a pressing need to address the harmonization of the teaching of posterior restoration as part of a global collaborative approach in phasing out the use of amalgam in this part of the world.

## 5. Conclusions

There are notable variations and diversities in the teaching of posterior restoration which shows a lack of consensus and agreement among dental schools. Therefore, there is a pressing need for uniformity, harmonization and consistency in the approaches used in primary dental qualification curricular. This action demands a collaborative effort by the dental schools in Malaysia. This study can be considered as an initial initiative in acquiring comprehensive information with regarding the operative dentistry teaching curriculum in dental schools in Malaysia. The global approach in phasing out the use of dental amalgam should not be ignored.

## Figures and Tables

**Table 1:** Teaching strategies for posterior restoration (*n* = 23).

Teaching Strategies	N (%)
Mode of Delivery (Core)	
Formal lecture	22 (95.7)
Demonstration	20 (87.0)
Problem-based learning (PBL)	17 (73.9)
Tutorial	16 (69.6)
Seminar	11 (47.8)
Material (Supplementary)	
Instruction manual	19 (82.6)
Models	15 (65.2)
Projection slides	8 (34.8)
Video tape	5 (21.7)
Overhead projector	1 (4.3)
Assessment methods	
Continuous assessment	19 (82.6)
Practical competency test	19 (82.6)
Written paper	16 (69.6)
Self-assessment	10 (43.5)
Peer assessment	8 (34.8)
Objective structural practical exam	7 (30.4)
Objective Structured Clinical Examination (OSCEs)	1 (4.3)

**Table 2:** The mean number of teeth needed for preclinical simulation laboratory practice (*n* = 23).

Type of Cavity	Type of Restoration							
	Amalgam				Composite			
	Range	Premolar	Range	Molar	Range	Premolar	Range	Molar
		Mean (±SD)		Mean (±SD)		Mean (±SD)		Mean (±SD)
Shallow cavity	0–3	1.09 (±0.85)	0–4	1.43 (±0.93)	0–3	1.26 (±0.81)	0–3	1.29 (±0.78)
Moderate cavity	1–4	1.70 (±0.77)	-	-	1–3	1.52 (±0.67)	1–3	1.48 (±0.68)
Deep cavity	0–4	1.52 (±0.95)	1–4	1.67 (±0.86)	0–3	1.48 (±0.79)	1–3	1.43 (±0.60)

**Table 3:** The use of liners/base prior to the placement of restoration in Malaysia dental schools (*n* = 24).

Cavity Depth	Type of Restoration	
	Amalgam	Composite
	N (%)	N (%)
Moderate		
No liner/base	11 (45.8)	12 (50.0)
GIC (base)	8 (33.3)	9 (37.5)
Hard-setting calcium hydroxide (liner)	8 (33.3)	13 (54.2)
Hard-setting calcium hydroxide (liner) + GIC base	11 (45.8)	7 (29.2)
Deep		
No liner/base	4 (16.7)	4 (16.7)
GIC (base)	6 (25.0)	8 (33.3)
Hard-setting calcium hydroxide (liner)	7 (29.2)	9 (37.5)
Hard-setting calcium hydroxide (liner) + GIC base	22 (91.7)	19 (79.2)

**Table 4:** Restorative materials taught for posterior tooth (*n* = 24).

Posterior Sites	Type of Restoration		
	Amalgam	Composite	GIC
	N (%)	N (%)	N (%)
Recommended			
Maxillary premolar	7 (29.2)	22 (91.7)	1 (4.2)
Maxillary molar	18 (75.0)	18 (75.0)	1 (4.2)
Mandibular premolar	7 (29.2)	23 (95.8)	1 (4.2)
Mandibular molar	18 (75.0)	17 (70.8)	1 (4.2)
Commonly placed			
Maxillary molar	15 (62.5)	15 (62.5)	2 (8.3)
Mandibular premolar	5 (20.8)	20 (83.3)	2 (8.3)
Mandibular molar	14 (58.3)	16 (66.7)	2 (8.3)
Maxillary premolar	5 (20.8)	20 (83.3)	2 (8.3)

**Table 5:** Taught finishing technique/materials for posterior restoration taught in Malaysia (*n* = 24).

Instruments/Devices	N	%
Diamond burs	11	45.8
Tungsten carbide (TC) burs	14	58.3
Finishing discs	21	87.5
Finishing strips	18	75.0
With water cooling	20	80
Without water cooling	5	20

## Supplementary Materials

The following are available online at https://www.mdpi.com/article/10.3390/dj9100123/s1, Figure S1: Teaching of Posterior Composite Resin Restorations in Undergraduate Dental Schools in Malaysia.
