# Supplementary material for: A Contemporary Evaluation on Posterior Direct Restoration Teaching among Undergraduates in Dental Schools in Malaysia
Source: Dent J (Basel). 2021 Oct 19;9(10):123. doi: 10.3390/dj9100123 (PMC8534716; doi:10.3390/dj9100123)
Supplement: Supplementary file 1 [file dentistry-09-00123-s001.zip › dentistry-1384323-supplementary.pdf]

Figure S1

TITLE:
TEACHING OF POSTERIOR COMPOSITE RESIN RESTORATIONS IN UNDERGRADUATE DENTAL SCHOOLS IN MALAYSIA

This questionnaire is designed to determine the details of posterior restorations curriculum for undergraduate teaching in your school. Please tick in the box provided.

Part A: SOCIAL DEMOGRAPHIC.

Q1. Please indicate your University & Department

Name of the University and Department: ____

Part B: TEACHING METHODS FOR POSTERIOR RESTORATIONS

Please tick in the box provided. For each statement, you can choose more than one answer.

Q1. Are formal lectures on posterior restoration topics given to students?

Yes ☐ No ☐

Q2. Other than formal lectures, other method(s) for teaching is/are:

Seminar ☐
Tutorial ☐
Problem based learning (PBL) ☐
None ☐
Other: ______________________________

Q3. Choose the teaching aids and materials that are used for teaching:

Models	☐	Demonstrations	☐	Instruction manuals	☐
Video tapes	☐	Transparencies	☐	Projection slides	☐
Hand-outs	☐	Posters	☐	None	☐

Other: ____

Q4. What are the type of assessments conducted to evaluate the students' knowledge on posterior restorations:

Continuous assessment ☐
Written paper ☐
Practical competency test ☐
Case report ☐
Objective structural practical examination (OSPE) ☐
Self-assessment ☐
Peer-assessment ☐
Other: ____

Part C: PRECLINICAL LABORATORY TEACHING ON POSTERIOR RESTORATIONS

Please tick in the box provided.

Q1. Do the students need to undergo preclinical teaching of posterior restorations before treating patient?

Yes ☐ No ☐

If the answer for Q1 is NO, please proceed to **Part D**.

Q2. Restorative material(s) used for preclinical teaching of posterior restorations is/are:
You can tick more than one answer.

Amalgam ☐
Composite resin ☐
Glass ionomer cement (GIC) ☐
Resin modified glass ionomer cement (RMGIC) ☐
Other: ____

Q3. Duration of time (hours) that is allocated is:
Please tick only **ONE (1)** answer for each restorative material.

**Restorative material**	**4–8 hours (1-2 sessions)**	**12–16 hours (3-4 sessions)**	**20–24 hours (5-6 sessions)**	**More than 24 hours (more than 7 sessions)**	**Not taught**
Amalgam					
Composite					
Glass ionomer cement					

Q4. Students are provided with instruction manual:

Yes ☐ No ☐

If the answer for Q3 is NO, please proceed to **Q6**.

Q5. Instruction manual provided to the students is generally:
Please tick only **ONE (1)** answer.

Simple (A basic guideline for posterior restorations) ☐
Comprehensive (Details in every procedure in posterior restorations) ☐
Very comprehensive (Could be used in place of textbook) ☐
Other: ____

Q6. During the preclinical session, students are using:
Please tick only **ONE (1)** answer.

Extracted human teeth ☐
Plastic tooth practice model ☐
Both ☐
Other: ____

Q7. Do the students need to pass the ***practical competency test*** before they start the clinical session?

a. Proximal amalgam restoration Yes ☐ No ☐
b. Proximal composite restoration Yes ☐ No ☐

Q8. Do the students have to complete certain ***practical requirement*** before they start the clinical session?

a.	Proximal amalgam restoration	Yes	☐	No	☐
b.	Proximal composite restoration	Yes	☐	No	☐

Q9. Please indicate the number of **premolar teeth** used for preclinical teaching of posterior restorations. Please tick only **ONE (1)** answer for each cavity depth.

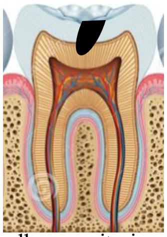

Shallow cavity involving outer 1/3rd dentine

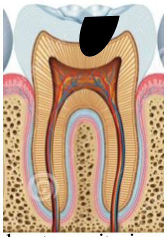

Moderate cavity involving middle 1/3rd dentine

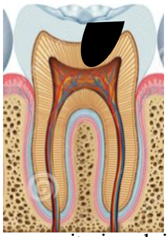

Deep cavity involving inner 1/3rd dentine

Material	Cavity depth	0	1	2	3	4	5	More than 5
**Amalgam**	Shallow occlusal cavity							
	Moderate occlusal cavity							
	Deep occlusal cavity							
**Composite**	Shallow occlusal cavity							
	Moderate occlusal cavity							
	Deep occlusal cavity							

Q10. Indicate the number of **molar teeth** used for preclinical teaching of posterior restorations. Please tick only **ONE (1)** answer for each cavity depth.

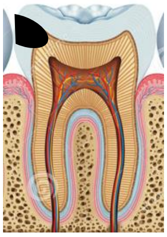

Shallow cavity involving outer 1/3rd dentine

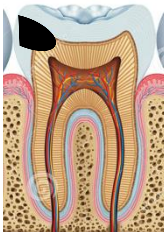

Moderate cavity involving middle 1/3rd dentine

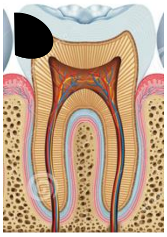

Deep cavity involving inner 1/3rd dentine

Material	Cavity depth	0	1	2	3	4	5	More than 5
**Amalgam**	Shallow proximal cavity							
	Moderate proximal cavity							
	Deep proximal cavity							
**Composite**	Shallow proximal cavity							
	Moderate proximal cavity							
	Deep proximal cavity							

Part D: CLINICAL TEACHING, TRAINING & ASSESSMENT ON POSTERIOR RESTORATIONS.

Q1. Teaching on the use of **liners and bases** for posterior composite restorations:
You can tick more than one answer.

Restorative material	Restorative protocols	Moderate cavity	Deep cavity
**Amalgam**	GIC (base)		
	Hard setting calcium hydroxide (liner)		
	Hard setting calcium hydroxide (liner) + GIC (base)		
	No liner and/or base		
**Composite**	GIC (base)		
	Hard setting calcium hydroxide (liner)		
	Hard setting calcium hydroxide (liner) + GIC (base)		
	No liner and/or base		

Q2. Which restorative material(s) is/are recommended for posterior sites?
You can tick more than one answer.

Restorative material	Amalgam	Composite	GIC
Maxillary premolar			
Mandibular premolar			
Maxillary molar			
Mandibular molar			

Q3. Which restorative material(s) is/are most commonly placed at posterior sites by students?
You can tick more than one answer.

Restorative material	Amalgam	Composite	GIC
Maxillary premolar			
Mandibular premolar			
Maxillary molar			
Mandibular molar			

Q4. What is/are the standard **isolation method(s)** taught for posterior restorations?
You can tick more than one answer.

Isolation technique	Rubber dam	Cotton roll	No isolation required
**Amalgam**			
**Composite resin**			
**Glass ionomer cement**			

Q5. Do you think that the teaching and use of posterior amalgam restorations is still relevant?
Please tick only **ONE (1)** answer.

Highly relevant	
Possibly relevant	
Possibly not relevant	
Not relevant	

Q6. What is the standard **restorative technique** taught for posterior composite restorations? You can tick more than one answer.

**Restorative technique**	Bulk fill	Incremental fill
**Shallow cavity**		
**Moderate cavity**		
**Deep cavity**		

Q7. Please indicate the **restorative protocol(s)** taught for posterior composite restorations? You can choose more than one answer for each statement.

**Criteria**	**Restorative protocols**	**Yes**	**No**
**Bevelling**	Bevel occlusal margins		
	Bevel the proximal box margins		
	No bevel		
**Matrix and wedging techniques (for proximal cavities)**	Circumferential metal band with wooden wedge		
	Sectional metal band with wooden wedge		
	Transparent matrix band with a light-transmitting wedges		
	Sectional matrix system (Palodent)		
**Bonding system**	Total-etch system		
	Self-etch system		
**Curing lights used**	Quartz-halogen light		
	Light emitting diode (LED) light		
	Both lights		
**Finishing techniques**	Finishing with water cooling		
	Finishing without water cooling		
**Finishing instruments**	Finishing diamond burs		
	Finishing TC burs		
	Finishing discs		
	Finishing strips		

Q8. Do the final year students have to complete certain clinical requirement for posterior restorations before they are allowed to sit for final examination?

**Restorative Material**	**Yes**	**No**
Amalgam		
Composite		

Q9. Please indicate the assessment/s conducted to evaluate the students' clinical competency? You can choose more than one answer.

Clinical viva	
Clinical competency test	
Objective structural clinical examination (OSCE)	
Self-assessment	
Peer-assessment	

Other: ____

Q10. Do you anticipate any changes to this aspect of your curriculum in the next year or two based on the new literature findings?

____

____

____

Thank you most sincerely for completing this questionnaire.
